# Conducting process evaluations of WASH interventions: a scoping review of design approaches and indicators in low- and middle-income countries

**DOI:** 10.1186/s43058-026-00872-8

**Published:** 2026-02-17

**Authors:** Jedidiah S. Snyder, Erika Canda, Lilly A. O’Brien, Erin E. Reardon, Emily A. Ogutu, Sara Hoffman, Paul Bagtas, Florence Santos, Matthew C. Freeman

**Affiliations:** 1 Gangarosa Department of Environmental Health, Rollins School of Public Health, Emory University, 1518 Clifton Rd, Atlanta, GA 30322 USA; 2 Woodruff Health Sciences Center Library, Emory University, Atlanta, GA USA; 3 CARE, Atlanta, GA USA

## Abstract

**Background:**

Process evaluation is a key methodological approach in implementation science, used to examine how interventions are delivered, adapted, and experienced in real-world contexts. While widely applied in clinical and public health settings, its use in the water, sanitation, and hygiene (WASH) sector is less documented. This review synthesized process evaluation methods used in WASH interventions in low- and middle-income countries (LMICs) to inform more systematic, framework-guided, and actionable implementation-focused evaluations.

**Methods:**

We systematically searched five electronic databases (Ovid MEDLINE, EMBASE, Scopus, Web of Science, and Global Health) and conducted outreach to researchers and practitioners to identify additional peer-reviewed and grey literature. Eligible studies included process evaluations or fidelity assessments of WASH interventions in LMICs, published from January 2008 onward. Data were extracted on study characteristics, use of conceptual or theoretical frameworks, methods, data types, and process indicators mapped across standard domains.

**Results:**

Twenty-four studies were included, spanning diverse WASH interventions. Sixteen studies used a conceptual or theoretical framework to guide process evaluation. Most (63%) used both quantitative and qualitative methods, while 29% used quantitative and 8% used qualitative methods alone. Process evaluations utilized multiple data types, including programmatic data, household surveys, qualitative interviews, focus group discussions, and structured observations. We identified 33 quantitative and 16 qualitative process indicators across 6 domains—fidelity, dose delivered, dose received (exposure), dose received (satisfaction), reach, and recruitment—to facilitate synthesis and identify patterns in how implementation was assessed. Quantitative approaches most frequently assessed fidelity, dose delivered, dose received (exposure), and reach, while qualitative approaches explored dose received (satisfaction) and recruitment.

**Conclusions:**

Process evaluation is increasingly used to strengthen the design and delivery of WASH interventions, yet variation persists in how implementation is defined, measured, and reported. More intentional application of frameworks and structured reporting can enhance the clarity and comparability of implementation assessments, including more systematic approaches for describing context. By consolidating quantitative and qualitative indicators and the data types used to measure them across 6 domains, this review provides a foundation to support more transparent, standardized, and framework-guided process evaluations that can strengthen learning, adaptation, and implementation practices in the WASH sector.

**Supplementary Information:**

The online version contains supplementary material available at 10.1186/s43058-026-00872-8.

Contributions to the literature

- This review extends the application of implementation science to the WASH sector by synthesizing process evaluation methods used across 24 WASH interventions in LMICs.
- It identifies how quantitative and qualitative methods align with specific process evaluation domains, informing more deliberate selection of methods to match implementation research questions.
- It contributes a synthesized set of process indicators and practical examples to support more systematic, framework-guided assessment of implementation processes in WASH interventions.
- It underscores opportunities to integrate process evaluation into routine program cycles and enhance implementation measurement and reporting by applying established implementation science frameworks more consistently.

## Background

Process evaluation is a methodological approach used to document intervention implementation and delivery. Rather than focusing solely on results, process evaluations examine the intervention design, services delivered, populations reached, resources used, and challenges encountered and addressed during implementation. It enables stakeholders to assess why an intervention achieved—or failed to achieve—its intended outcomes [1–5]. This contrasts with outcome or impact evaluations, which assess program effectiveness or efficacy by determining whether and how targeted outcomes were achieved [6]. Process evaluations provide feedback on the extent to which an intervention is being delivered as it was originally planned or intended (i.e., implementation fidelity) [7], identify barriers [1–5], and inform necessary adaptations [8]. Most importantly, they help explain why interventions succeeded or failed to achieve their intended benefits, strengthening the evidence base for replication, scale-up, and policy formulation [9].

In the water, sanitation, and hygiene (WASH) sector, numerous interventions have been implemented over the past several decades to improve public health [10, 11], but an unknown number have reported on process measures. A 2021 Campbell systematic review on the effectiveness of WASH interventions identified at least 367 completed or ongoing impact evaluations in low- and middle-income countries (LMICs), nearly three-quarters of which were conducted after 2008, along with 43 systematic reviews [12]. While the quality of impact evaluations has improved over time—for instance, through the adoption of rigorous study designs, efforts to minimize reporting bias, and the use of sufficiently powered cluster samples—progress in documenting how interventions were implemented has remained relatively limited [12]. A review of the forty most cited evaluations of WASH interventions published between 2012–2022 found that implementation fidelity was not reported in over a third of reviewed articles, including their supplements, protocols, and other associated materials [13]. This highlights a persistent gap that implementation science tools like the Template for Intervention Description and Replication for WASH (TIDieR-WASH) checklist aim to address by standardizing the documentation of key implementation elements to improve transparency, replicability, and learning across WASH programs [14]. Although TIDieR-WASH is not a process evaluation framework, it applies core implementation science principles by providing a structured approach for reporting implementation details, such as fidelity, intervention dose (i.e., the quantity of an intervention delivered and amount of participation), and adaptations, that are central to process evaluation. Consistent use of TIDieR-WASH can therefore strengthen the quality and completeness of implementation reporting in WASH studies, helping to address the persistent gaps identified in recent evaluations.

The lack of process evaluations, and their reporting, makes it difficult to understand whether suboptimal health outcomes stem from issues related to implementation, underlying theory, or a combination of both [15]. Programmatic learning, central to implementation science, supports real-time improvement of interventions and enhances accountability by helping stakeholders understand and demonstrate value for money [15]. Lessons from recent implementation research in LMICs emphasize that, while process evaluations may require careful planning and coordination, they significantly enhance understanding of the factors that influence implementation of complex interventions [16]. This research also underscores the value of initiating process evaluations early in the project lifecycle to inform intervention design, as well as the importance of strong communication between teams responsible for implementation, process evaluation, and outcome evaluation. In contrast, collecting process evaluation data only after the intervention has ended limits the ability to meaningfully assess how implementation occurred [17].

Process evaluation is an important component of evaluating complex interventions [3, 5]. Yet, there has been little work to understand how process evaluations are conducted in the WASH sector, particularly in LMICs. A scoping review of methodologies used in process evaluations of WASH interventions in LMICs is critical for several reasons. First, it addresses a knowledge gap by synthesizing the diverse approaches used to assess intervention implementation. Such a review provides a comprehensive understanding of current approaches and offers insights into how they have been applied across different contexts. Furthermore, the findings from this review may help inform future evaluation efforts by providing practitioners and researchers with a clearer picture of how process evaluations have been applied in the WASH sector. This information can support more contextually appropriate and practical approaches to assessing implementation, particularly in resource-constrained settings.

## Objectives

This scoping review aims to map how process evaluations are applied in the context of WASH interventions in LMICs. Specifically, it seeks to provide a broad understanding of the methodologies used to evaluate intervention delivery in development settings where programs are implemented across diverse contexts, involve multiple actors, and require adaptive approaches [15]. The intent of this review is not to assess the effectiveness or outcomes of process evaluations themselves, but rather to describe how they have been designed and applied. This focus is consistent with the purpose of scoping reviews, which aim to map the existing evidence base and identify patterns, gaps, and areas for future research rather than evaluate intervention impacts.

The specific objectives are to: (1) Examine how process evaluations are designed and conducted in WASH interventions, including frameworks, methods, and data types used; and (2) Identify and synthesize indicators used in process evaluations of WASH interventions, including how these are applied across standard process evaluation domains.

## Methods

We structured our study using the Preferred Reporting Items for Systematic reviews and Meta-Analyses extension for Scoping Reviews (PRISMA-ScR) Checklist [18] (See Supplemental Material – PRISMA-ScR Checklist) and methodological guidance for scoping studies [19, 20].

### Eligibility criteria

We included process evaluations of randomized controlled trials (RCTs) and non-RCTs, including feasibility studies, observational studies, and quasi-experimental studies. Eligible studies were either explicitly labeled as process evaluations or had a stated aim of understanding the extent to which intervention activities were implemented as planned (i.e., implementation fidelity). We limited inclusion to studies in non-health care settings to focus on process evaluations relevant to community-based WASH programs, where delivery strategies and implementation actors differ substantially from clinical environments. Interventions examined in the included studies involved any combination of WASH components, such as improving drinking water quality or access, reducing exposure to human feces through sanitation systems, promoting hand hygiene at key moments, and addressing menstrual and food hygiene practices. Studies published from January 2008 onward were included, consistent with the year the Medical Research Council (MRC) updated its guidance on developing and evaluating complex interventions (with a subsequent update in 2021) [3, 5]. This timeframe captures the expanding use of process evaluations in implementation research along with an increasing volume of WASH-related studies published in literature [12]. Eligible studies were conducted in LMICs, as defined by the World Bank [21]. We excluded studies that were not based on empirical research, not reported in English, or were review articles.

### Information sources

We developed a comprehensive search strategy to identify relevant studies. We searched the following electronic databases for peer-reviewed literature: Ovid MEDLINE, EMBASE, Scopus, Web of Science, and Global Health. In addition to database searches, we contacted organizations implementing WASH programs, as well as researchers, to identify additional WASH-related process evaluations.

### Search strategy

The strategy was designed for Ovid MEDLINE in consultation with an informationist at Emory University (EER), using a combination of keywords and controlled vocabulary terms related to (1) process evaluations, (2) WASH interventions, and (3) exclusion criteria (i.e., non-human research). The search was then translated for use in the remaining four databases and conducted on May 15, 2024. A comprehensive list of search terms for each database is provided in Supplemental Material – Search Strategy.

### Selection of sources of evidence

**Fig. 1:**
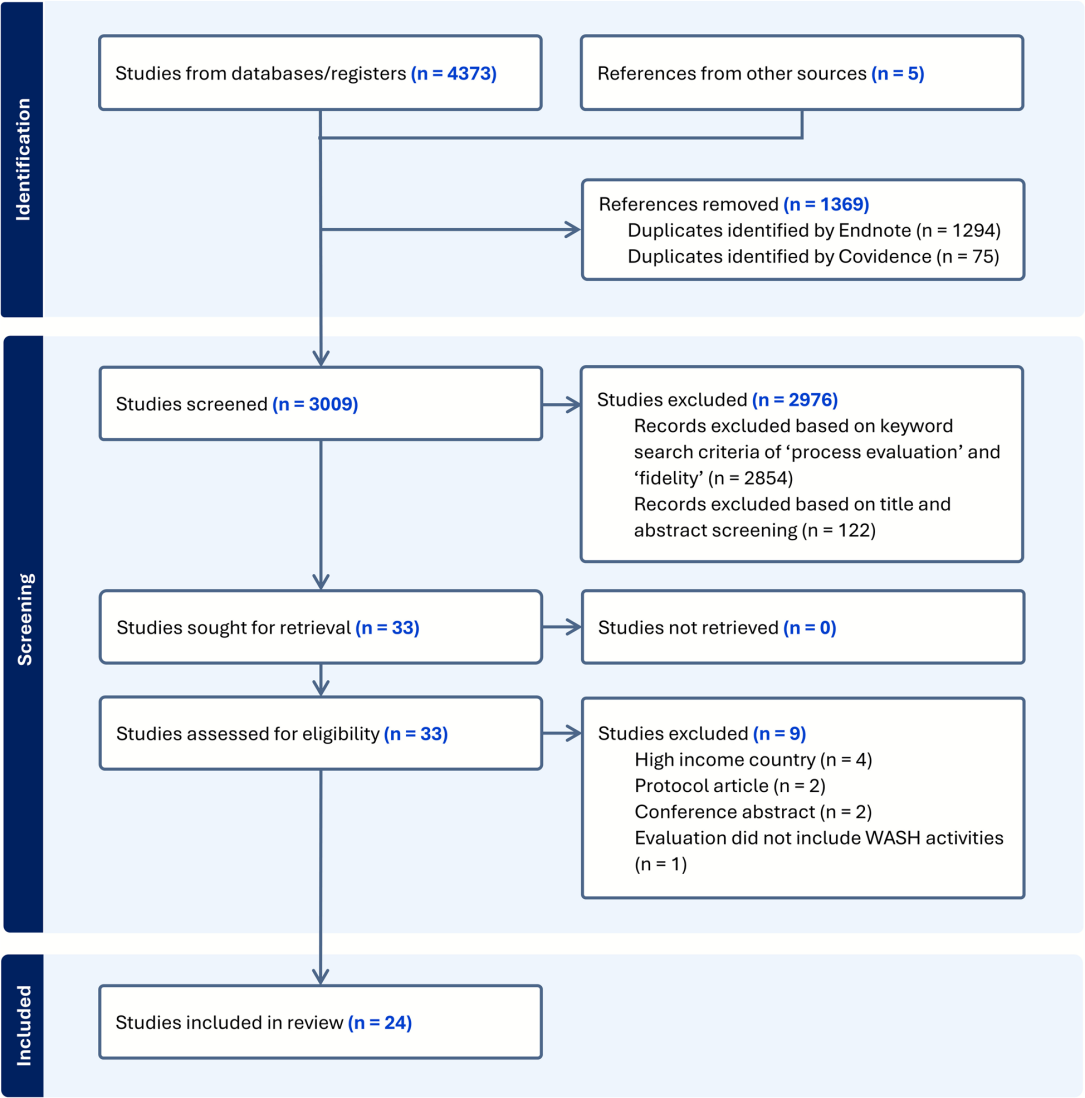
PRISMA flow diagram for the study selection process

Search results from each database were compiled, and duplicate records were removed in Endnote 21 [22] and Covidence [23]. To ensure a comprehensive search, broader terms such as 'program evaluation' and 'implementation assessment' were included in the initial strategy to capture the range of ways process evaluations may be described. However, for inclusion, we prioritized studies that explicitly used process evaluation terminology—'process evaluation' or 'fidelity'—in the title or abstract. This decision was made to ensure the inclusion of studies with detailed methodological reporting (i.e., details on frameworks, methods, data types, and indicators used to conduct the process evaluation) and to support a synthesis of clearly defined approaches that could offer more actionable guidance for WASH practitioners and researchers. Two reviewers (JSS, LAO) then independently screened the retained titles and abstracts based on the eligibility criteria. In cases of disagreement or uncertainty, articles were retained for full-text screening. The full texts of the remaining studies were retrieved and independently screened by both reviewers for eligibility. Covidence [23] was used as the primary screening tool. The stages of the search and screening process are described in Fig. 1.

### Data charting process

A standardized data extraction form was developed by the research team to systematically collect relevant information from the included studies. One reviewer (EC) independently extracted data from all included studies, with a second reviewer (JSS) supporting the resolution of uncertainties or discrepancies as needed. When supplemental materials were referenced but not available in the publication, we contacted study authors to request additional information to support data extraction. We did not conduct a critical appraisal of included sources of evidence, as the purpose of this review was to describe and characterize process evaluations rather than assess study quality or risk of bias.

### Data items

#### Characteristics of the study and WASH intervention

Data extraction captured key characteristics of the study and WASH intervention. This included the country, type of study (whether the process evaluation was part of a RCT or not), location (e.g., rural, urban), setting (e.g., households, schools), and target group of the intervention (e.g., community members, household members). We noted whether the study involved non-academic authors (based on authorship affiliations). The WASH intervention approach (demand-side, supply-side, or combined), their mechanism of delivery, and outcome type (behavioral or quality of life) were categorized based on standard definitions [12].

#### Details of the process evaluation

We systematically extracted details from each study’s process evaluation as reported in the methods section, including the stated aim, methodological approach (quantitative and/or qualitative methods), and associated data types. We recorded whether a guiding conceptual or theoretical framework was referenced for the process evaluation and, if so, documented the specific framework used. For each study, all clearly reported process indicators were extracted. These included *quantitative process indicators,* which refer to metrics to capture numerical data about implementation, such as *what* was delivered, *how often*, or *when* (e.g., number of visits conducted during the first implementation phase); and *qualitative process indicators,* which refer to the primary questions used to explore, understand, and interpret implementation processes through non-numerical data, such as *what* was implemented and *why or how* it occurred as it did (e.g., perceptions of activity quality or timing). Corresponding means of verification for indicators were also extracted, based on details reported in both the methods and results sections of each study.

### Synthesis of results

#### Objective 1: Examine how process evaluations are designed and conducted in WASH interventions, including frameworks, methods, and data types used

We present a descriptive summary of key study characteristics and WASH interventions included in this scoping review, including the type of intervention, the stated aim of the process evaluation, and the research methods used. We report on the number of studies that referenced specific conceptual or theoretical frameworks and describe their role in shaping evaluation design, data collection, or analysis. We synthesized the methods and data types used to assess implementation and categorized the timing of data collection.

Data types were grouped as quantitative (household surveys, structured observations, questionnaires, programmatic data, non-programmatic data) or qualitative (interviews, focus groups, semi/unstructured observations, programmatic documentation). Data types were categorized to reflect distinct data collection activities, rather than individual data elements. For example, although household surveys may incorporate observations or questionnaires, they were classified separately due to their role as a standalone data collection activity. Programmatic documentation was classified as qualitative when used to capture narrative data, implementation reflections, adaptations, or contextual insights (e.g., meeting notes, field reports, facilitator debriefs), rather than structured numeric indicators.

Although several included studies collected both qualitative and quantitative data, our categorization of 'data types' reflects the type of data collected, rather than the overall study design. Mixed-methods research is defined not merely by the concurrent use of qualitative and quantitative data, but by the intentional integration of these data strands through a clearly articulated methodological approach [24, 25]. Because mixed-methods is an overarching design strategy rather than a distinct type of data, we classified data types according to whether they produced qualitative or quantitative data, while noting when studies used both.

#### Objective 2: Identify and synthesize indicators used in process evaluations of WASH interventions, including how these are applied across standard process evaluation domains

To ensure consistency in reporting, study-specific process indicators were mapped to standard process evaluation domains—fidelity (quality), dose delivered (completeness), dose received (exposure), dose received (satisfaction), reach, recruitment—based on established definitions [1]. These domains included process indicators that were either explicitly labeled with the domain name (e.g., fidelity) by the study authors, or conceptually aligned with the domain’s definition—even when different terminology was used or when no domain was specified. The studies’ measurement of context—defined as aspects of the environment that may influence the implementation or outcomes of the intervention [1]—was not extracted or synthesized due to substantial variation in how context was defined, operationalized, and measured across studies. In many cases, context was described narratively or embedded within broader thematic findings, rather than reported as discrete, verifiable indicators.

Below are the standard definitions used to classify each process evaluation domain along with the ways in which these definitions were expanded to guide process indicators classification in this scoping review:

- Fidelity (quality): The extent to which the intervention was implemented as planned [1]. This also included indicators describing the structure, content, and quality of delivery.
- Dose delivered (completeness): The amount or number of intended units of each intervention component delivered or provided by interventionists [1]. This also included indicators related to the frequency, duration, and completion of tasks and activities.
- Dose received (exposure): The extent to which participants actively engaged with, interacted with, were receptive to, and/or used the materials or recommended resources; this can include both initial and continued use [1]. This also included indicators related to adoption, engagement, participation, and observed or reported responses to the intervention.
- Dose received (satisfaction): Participant (both primary and secondary audience) satisfaction with the program, including their interactions with staff and/or investigators [1]. This also included indicators related to acceptability, emotional responses, and suggestions for improvement.
- Reach (participation rate): The proportion of the intended target audience that participated in the intervention, often measured by attendance; this also includes documentation of barriers to participation [1]. This also included indicators related to participant demographics and reach by delivery channel.
- Recruitment: The procedures used to approach and attract participants at the individual or organizational level [1]. This also included indicators related to strategies used, challenges encountered, and factors supporting sustained engagement in intervention and evaluation activities.

Process indicators that measured similar aspects within a given domain were grouped across studies to facilitate synthesis and identify common patterns in how implementation was assessed. Quantitative and qualitative process indicators are reported separately to highlight their distinct contributions and to support their utility in guiding future applications.

## Results

### Characteristics of the studies included in this review

We identified 24 studies that met our inclusion criteria, including 21 journal articles, 2 studies published in grey literature, and 1 pre-print (Fig. 1). A summary of the included studies—covering the intervention descriptions, process evaluation aims, and methods used—is provided in Supplemental Material – Search Strategy. A descriptive summary of key study characteristics, WASH interventions, and methods applied in the process evaluations is presented in Table 1. The majority were conducted in the WHO African Region (67%), followed by the South-East Asian Region (29%) and the Western Pacific Region (8%), with one multi-country study (4%). Half of the studies were part of RCTs. Most interventions used demand-side approaches (87%), especially behavior change strategies. About half included supply-side components such as hardware provision (46%). Combined or integrated approaches were also common, including decentralization (4%) and mixed intervention mechanisms (38%). Reported intervention outcomes largely focused on behavioral change (83%), especially reduction in open defecation (71%), latrine use and maintenance (29%), and safe water treatment and storage (25%). Other outcomes included quality of life (33%), water-related illness (17%), and nutrition (17%). Non-academic partners were listed as co-authors in 63% of studies, suggesting meaningful collaboration between practitioners and researchers during the research process.

**Table 1:** Descriptive summary of key characteristics of the studies and WASH interventions

Descriptive characteristics of studies	n (24)	(%)
**WHO Region**		
African Region (AFR)	16	67%
South-East Asian Region (SEAR)	7	29%
Western Pacific Region (WPR)	2	8%
Multi-country	1	4%
**Process evaluation was part of a randomized trial**		
Yes	12	50%
No	12	50%
Intervention location*		
Rural	14	58%
Urban	3	13%
Peri-urban	3	13%
Unspecified	5	21%
Intervention setting*		
Household	16	67%
Community	9	38%
Schools	4	17%
Informal settlements	3	13%
Target group of the intervention*		
Household members	10	42%
Mothers/caregivers/children under 5 years old	10	42%
Community members	6	25%
School-age children (5–18 years old)	5	21%
Intervention approaches*		
Demand-side	21	87%
Behavior change intervention approaches	21	87%
Subsidies and microfinance	1	4%
Supply-side	12	50%
Direct hardware provision	11	46%
Improving operator performance	1	4%
Combined interventions	10	42%
Decentralization	1	4%
Combinations of intervention mechanism	9	38%
Intervention outcome categories*		
Behavioral	20	83%
Open defecation	17	71%
Construction, use, and maintenance of latrines	7	29%
Water treatment and storage practices	6	25%
Sustainability and slippage	4	17%
Water supply behavior	2	8%
Quality of life	8	33%
Water-related ill-health	4	17%
Nutrition	4	17%
**Non-academic partners listed as authors**		
Yes	15	63%
No	9	38%
**Conceptual or theoretical framework referenced in guiding process evaluation design**		
Yes	16	67%
No	8	33%
Methods/data types used*		
Quantitative	22	92%
Qualitative	17	71%
**Combinations of methods/data types used**		
Both quantitative and qualitative	15	63%
Quantitative only	7	29%
Qualitative only	2	8%

Note: Categories for descriptive characteristics marked with an asterisk (*) are not mutually exclusive; a single study may fall under multiple categories, resulting in percentages that exceed 100%

### Objective 1: Examine how process evaluations are designed and conducted in WASH interventions, including frameworks, methods, and data types used

#### Frameworks used in process evaluations of WASH interventions

Sixteen studies (67%) referenced a conceptual or theoretical framework to guide their process evaluation design (Table 1). The extent to which frameworks were explicitly used to guide indicator development or structure results varied, and several studies (n = 6, 25%) drew on more than one framework, as detailed in Supplemental Material – PRISMA-ScR Checklist. The most commonly applied framework was from the MRC guidance on process evaluations [3, 5], used in 8 studies (33%) to report on core domains such as fidelity, dose delivered and received, reach, acceptability, and contextual influences. *Process evaluation for public health interventions and research* [4] was identified in 7 studies (29%), primarily to assess fidelity, dose, reach, and recruitment. *A conceptual framework for implementation fidelity* [7] and *Developing a process-evaluation plan for assessing health promotion program implementation: a how-to guide* [1] were each explicitly used in 3 studies (13%), focusing on implementation fidelity, dose delivered, and reach. A smaller number of studies (n = 4, 17%) applied other frameworks, including the *Exploration, Preparation, Implementation, Sustainment (EPIS) framework* [26], *WHO’s orocess evaluation workbook* [27], and conceptual models for implementation outcomes and generalizability [28–30].

**Table 2:** Research methods and data types used in process evaluations of WASH interventions

Data type	Description	n (%)	Timing	Example application
Quantitative Methods				
Household surveys	Surveys conducted at the household level with intervention recipients to assess implementation by capturing data on exposure to intervention activities (e.g., receipt of materials or services), adoption of promoted practices or relevant behaviors (e.g., handwashing or latrine construction), and perceptions of the intervention	13 (54%)	Cross-sectionally during and after implementation	- Household surveys conducted by community health workers alongside educational visits to assess water filter adoption, maintenance, and training comprehension [31] - Household surveys conducted 4–6 weeks post-intervention to assess exposure, message recall, and perception shifts [32] - Endline household surveys assessed exposure to materials, participation in activities, and household-level intervention reach [33]
Structured observations	Direct, systematic observations assessing fidelity of delivery (i.e., whether intervention activities were conducted as intended) and the presence, functionality, and accessibility of intervention components experienced by end-users (e.g., WASH facilities, household materials)	12 (50%)	Routinely during implementation, immediately post-activity as needed	- Observations of WASH hardware functionality and handwashing in schools every 6–8 weeks [34] - Weekly unannounced observations of hygiene kit demonstrations and health promotion sessions [35] - Observations every 6–8 weeks of latrine construction progress in intervention villages [36]
Questionnaires	Questionnaire-based tools administered by evaluators—either digitally or in-person—to implementers (e.g., community health workers, teachers, volunteers) or to intervention recipients (e.g., caregivers, students) to assess training outcomes, message comprehension, awareness, perceptions, and self-reported behaviors	8 (33%)	Before and after specific activities or trainings	- Questionnaire collected via mHealth and Interactive Voice Response platforms to assess message recall and comprehension of health messages [37] - Questionnaire with nutrition promoters to assess training completion and message understanding [38] - Tablet-based questionnaire with students on menstrual health, product use, and sanitation perceptions [39]
Programmatic data	Program-generated data collected through routine monitoring forms, activity logs, or other tracking systems to document intervention delivery and assess participant exposure to key activities (e.g., session attendance, receipt of materials or services)	14 (58%)	Continuously during implementation	- Attendance and supply logs maintained by implementers to document key intervention events [40] - Program tracking data on attendance and service use, complemented by monitoring surveys and performance reviews of staff training and behavior change activities [38] - Community coordinators’ weekly reports documenting attendance, supply availability, and challenges encountered during intervention delivery and training sessions [41]
Non-programmatic data	Contextual or system-level data not generated by program activities but useful for understanding the broader environment in which an intervention is implemented (e.g., utility data, health surveillance data)	2 (8%)	Prior to and throughout implementation as needed	- Operational and billing records from a national water utility used to assess water system performance [42] - Government health surveillance data used to contextualize the intervention within broader public health trends [35]
Qualitative Methods				
Interviews	In-depth, semi-structured, or key informant interviews conducted with both implementers (e.g., program staff, community health workers) and intervention recipients (e.g., caregivers, community members) to explore experiences, perceptions, and contextual factors influencing intervention implementation	13 (54%)	During and after implementation	- Interviews with implementers, Ministry of Health staff, and volunteers on delivery roles and acceptability [43] - Interviews with household members on hygiene kit use and perceptions [35] - Interviews with school leaders and education officers exploring capacity to manage and maintain WASH infrastructure [44]
Focus group discussions (FGDs)	Group-based discussions conducted primarily with intervention recipients, and occasionally with implementers, to explore shared experiences, perceptions, and feedback on implementation, acceptability, and perceived impact	7 (30%)	During and after implementation	- FGDs with community residents on program engagement, participation barriers, and satisfaction [45] - FGDs with crisis-affected populations exploring the acceptability of a handwashing program and factors influencing engagement [46] - FGDs with local leaders and caregivers assessing acceptability of intervention messages, delivery methods, and cues to action [41]
Semi/ unstructured observations	Observations of intervention activities, participant behaviors, or implementation processes to document fidelity, capture engagement, and provide contextual insights not easily obtained through structured tools	4 (17%)	Opportunistically throughout implementation	- Observations during key program moments using semi-structured forms, photographs, and notes to assess fidelity and context [46] - Observations of training delivery, facilitator-participant interaction, use of materials, and engagement through checklists and free-form notes [43] - Fieldworkers' observations of intervention delivery with notes on challenges, deviations, and participant reactions, coded for acceptability, feasibility, and impact [32]
Programmatic documentation	Formal and informal program-generated documents—such as reports, field notes, debriefs, meeting minutes, photographs, and reflections—used to retrospectively document implementation processes, surface challenges, and capture contextual insights	11 (46%)	Prior to and throughout implementation, at key milestones	- Meeting minutes and field notes reviewed to track coordination and emerging issues [47] - Government reports and supervision memos reviewed for school WASH implementation [44] - Debriefing notes reviewed to capture challenges and contextual barriers [48]

#### Methods used in process evaluations of WASH interventions

Of the 24 included studies, 92% used quantitative methods and 71% used qualitative methods, with considerable overlap between the two. Specifically, 63% employed both quantitative and qualitative methods, 29% used only quantitative methods, and 8% relied solely on qualitative methods (Table 1). Importantly, the concurrent use of qualitative and quantitative methods does not necessarily constitute a mixed-methods design, which requires intentional integration rather than simple co-occurrence. These findings therefore reflect the range of methods employed across studies and highlight the predominant use of quantitative approaches, alongside frequent incorporation of qualitative approaches to broaden understanding of implementation.

#### Data types used in process evaluations of WASH interventions

Various types and combinations of data were used to support process evaluations of WASH interventions in the included studies (Supplemental Material – Search Strategy). A summary of the data types categorized by quantitative and qualitative approaches is provided in Table 2. This table presents a description of each data type as applied within WASH process evaluations, along with illustrative examples from included studies demonstrating their use. The most applied data types were quantitative programmatic data (58%) and household surveys (54%), and qualitative interviews with intervention stakeholders (e.g., implementers, government representatives, community leaders, and intervention recipients) (54%). Programmatic data—such as attendance records, session logs, and monitoring reports—were widely leveraged to document intervention delivery and participant exposure. Household surveys provided structured insights into participant experiences, exposure to intervention activities, and behavioral uptake. Interviews with implementers and recipients enabled deeper exploration of delivery processes, barriers encountered, and factors influencing outcomes. Structured observations were also widely used (50%) to collect quantitative data for objective assessment of implementation fidelity, participant engagement, and the presence and condition of intervention components such as WASH infrastructure and materials. Use of qualitative programmatic documentation (46%) and focus group discussions (30%) was common to capture insights on implementation processes and shared experiences of intervention stakeholders. Questionnaires (33%) were used to quantitatively assess knowledge, behaviors, and implementation fidelity among recipients and implementers. Less frequently, semi- or unstructured observations (17%) were used qualitatively to capture insights of engagement and fidelity in real-time, while non-programmatic data (8%) were used to supplement quantitative findings with external contextual information (e.g., utility data, health surveillance data).

### Identify and synthesize indicators used in process evaluations of WASH interventions, including how these are applied across standard process evaluation domains

**Fig. 2:**
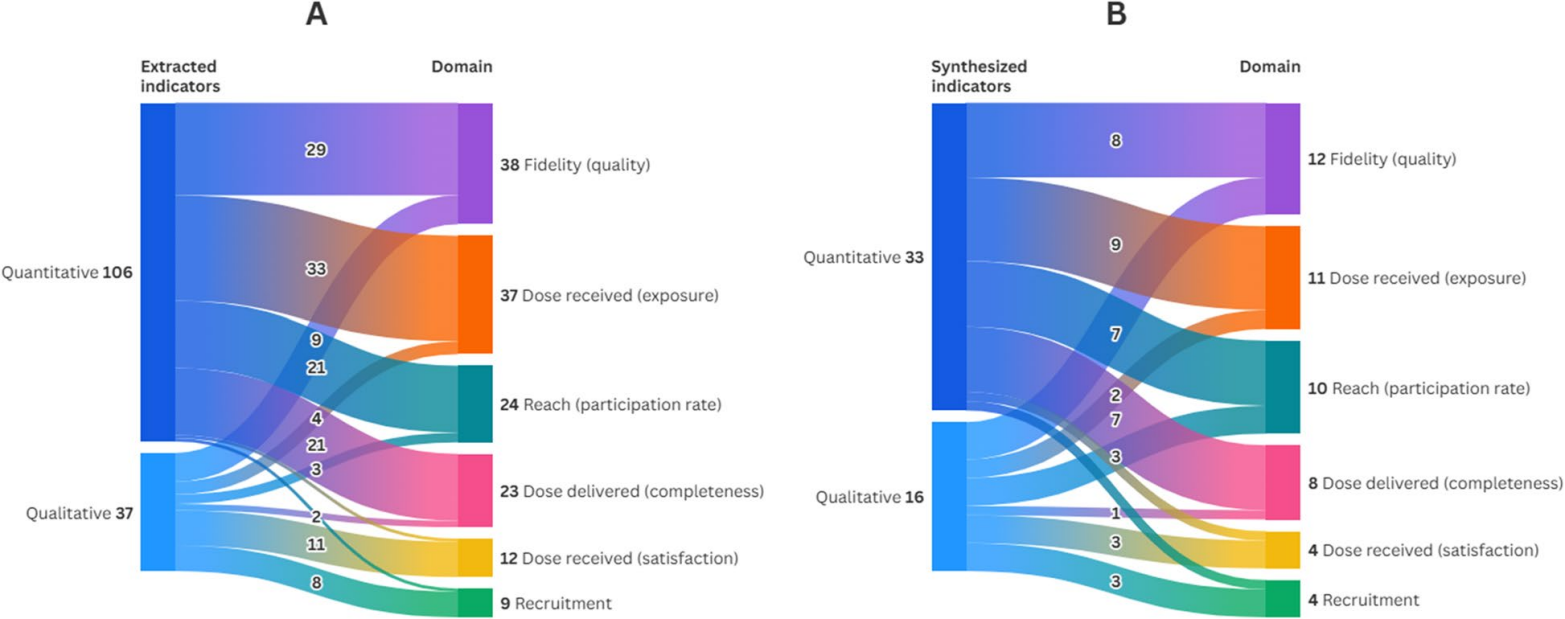
Mapping of extracted and synthesized process indicators across domains. Panel A presents the extracted indicators mapped to their corresponding process evaluation domains, while Panel B displays the synthesized indicators mapped to domains following consolidation across studies

Across the included studies, we extracted 106 quantitative and 37 qualitative indicators reflecting how implementation was assessed across the WASH process evaluation literature. When organized by the 6 core process evaluation domains, these indicators encompassed 33 distinct quantitative and 16 distinct qualitative measures. Figure 2 illustrates how these indicators map across domains, and Tables 3 and 4 provide detailed descriptions, means of verification, and references to the studies with each indicator appeared.

**Table 3:** Quantitative process indicators used in process evaluations of WASH interventions

#	Quantitative process evaluation indicators	Means of verification	Study references	Number of references
**1. Fidelity (quality):** Extent to which intervention was implemented as planned				**n = 29**
1.1	**Infrastructure delivery quality:** Number or proportion of hardware, software, consumables, and services delivered, received, and used as planned (e.g., latrines built, soap distributed, water supply connections installed, functional handwashing stations)	Structured observations of WASH facilities; questionnaire with recipients; programmatic data (voucher logbook, activity log, stock sheet); non-programmatic data (operational records)	[33, 34, 36, 38–40, 42, 47, 49]	9
1.2	**Behavior change delivery quality:** Number or proportion of behavior change activities conducted as planned (e.g., skits, hygiene sessions, WASH club meetings)	Programmatic data (activity log); household surveys; structured observations during activities	[33, 39–41, 48, 49]	6
1.3	**Timely completion of planned sessions:** Number or proportion of sessions completed within the intended timeframe and with planned content (e.g., household visits completed on schedule, weekly community meetings held, training modules completed)	Programmatic data (activity reports); structured observations during activities; household surveys	[33, 38, 39, 41, 49]	5
1.4	**Activity delivery quality:** Number or proportion of activities that met delivery standards in terms of quality, efficiency, and content (e.g., use of appropriate materials, adherence to facilitator protocols, correct demonstration techniques)	Structured observations during activities; programmatic data (activity logs)	[38, 40, 41]	3
1.5	**Message delivery quality:** Number or proportion of program messages delivered and received as planned (e.g., handwashing messages conveyed during school sessions, posters displayed in designated locations, SMS reminders sent)	Programmatic data (surveillance forms); structured observations during activities	[37, 38, 49]	3
1.6	**Implementer adherence:** Number or proportion of program implementers demonstrating intended knowledge of program components (e.g., correctly describing handwashing steps, explaining latrine maintenance protocols, using visual aids appropriately)	Questionnaire assessing implementer knowledge	[38]	1
1.7	**Gender-responsive planning:** Number or proportion of sex-segregated activities conducted as planned (e.g., sessions led by female facilitators for women participants, separate youth group discussions)	Structured observation during activities; programmatic data (activity logs)	[49]	1
1.8	**Inclusive planning:** Number or proportion of suitable venues used for activities as planned (e.g., accessible meeting spaces for people with disabilities, venues near target populations, private venues)	Programmatic data (community records)	[49]	1
**2. Dose delivered (completeness):** Amount or number of intended units of each intervention or component delivered or provided by interventionists				**n = 21**
2.1	**Training delivery:** Number or proportion of training sessions or modules conducted (e.g., staff or volunteer capacity-building sessions)	Questionnaire with implementers; structured observations of training activities; programmatic data (activity checklists, school attendance logs, school reports)	[38, 39, 41, 50, 51]	5
2.2	**Message dissemination:** Number or proportion of health promotion messages delivered through various media (e.g., billboards, community campaigns)	Interviews with implementers and recipients; structured observations of activities; programmatic data (activity and performance reports)	[33, 35, 38]	3
2.3	**Household visits:** Number or proportion of initial and follow-up household visits conducted (e.g., behavior change reinforcement, counseling visits)	Household surveys; programmatic data (summary reports, household calendar)	[41, 48, 51]	3
2.4	**Implementation staff activity completion:** Number or proportion of implementation staff completing intervention activities (e.g., forming community groups, completing participant tracking forms, delivering school lessons)	Questionnaire assessing implementer knowledge; programmatic data (program reports, school attendance and observation logs)	[39, 50, 51]	3
2.5	**Material delivery:** Number or proportion of consumables and infrastructure components distributed or installed (e.g., soap, hygiene kits, nutrition supplements)	Household surveys; programmatic data (reports, activity logs)	[35, 38, 39]	3
2.6	**Community activity execution:** Number or proportion of community intervention activities implemented (e.g., community health club sessions, community events, school performances)	Programmatic data (activity checklists, school activity logs, community records, attendance logs, performance reports)	[39, 49, 51]	3
2.7	**Activity duration:** Length of intervention activities per session (e.g., total minutes/hours of each training session or community activity)	Structured observations of activities	[51]	1
**3. Dose received (exposure):** Extents to which participants actively engage with, interact with, are receptive to, and/or use materials or recommended resources; can include “initial use” and “continued use”				**n = 33**
3.1	**Household visit exposure and frequency:** Number or proportion of household members who reported receiving or recalling a visit, and their reported frequency/timing of visits (e.g., weekly caregiver check-ins, monthly compound health visits)	Household surveys; questionnaires with recipients; programmatic data (summary reports, household visit records)	[31, 36, 38, 41, 45, 48, 49, 52–54]	10
3.2	**Material presence, use, and condition:** Number or proportion of households or schools displaying, using, or having present and functional intervention items (e.g., visible posters, installed handwashing stations with water and soap, working water filters)	Structured observation of WASH facilities and consumables; household surveys	[31, 32, 38, 39, 41, 44, 48, 54]	8
3.3	**Campaign recall:** Number or proportion of participants who recalled intervention-related campaigns (e.g., radio jingles on safe water, SMS messages on hygiene, community posters)	Household surveys; programmatic data (web-based mobile message platform)	[32, 36–38]	4
3.4	**Message recall:** Number or proportion of participants demonstrating knowledge and recall of key intervention personnel, elements, and messages (e.g., names of facilitators, benefits of handwashing, key messages from trainings)	Household surveys; questionnaires with participants on program knowledge and recall	[37, 41, 51]	3
3.5	**Counseling visit discussion recall:** Number or proportion of households recalling specific topics discussed during visits and the frequency with which they were mentioned (e.g., latrine construction, handwashing, water treatment)	Household surveys; questionnaires on intervention delivery with recipients	[36, 51, 53]	3
3.6	**Community activity recall:** Number or proportion of participants who recalled knowledge or awareness of community clubs, committees, or events (e.g., village water and sanitation committee meetings, community-led total sanitation walks)	Questionnaires of knowledge and awareness of activities with implementors and recipients	[36, 51]	2
3.7	**Gaps in supplies or services:** Number or proportion of households reporting gaps in access to or presence of intervention supplies or services, and the average duration of those gaps (e.g., days without soap at handwashing station, weeks without access to water filters)	Household surveys	[38]	1
3.8	**WASH group affiliation:** Number or proportion of households with a WASH group membership (e.g., registered in village WASH committee, active member of local sanitation club)	Household surveys	[52]	1
3.9	**Staff participation and recall of training/meetings:** Number or proportion of implementation staff or volunteers who were invited to, attended, and recalled trainings or committee meetings, including meeting frequency and topics (e.g., invited to monthly team meetings, remembered hygiene campaign goals, attended refresher trainings)	Questionnaire of knowledge and awareness of activities with implementors	[36]	1
**4. Dose received (satisfaction):** Participant (primary and secondary audiences) satisfaction with program, interactions with staff and/or investigators				**n = 1**
4.1	**Participant satisfaction:** Level of satisfaction among participants (e.g., satisfaction with intervention delivery, perceptions of staff courtesy, ease of accessing services)	Household surveys	[45]	1
**5. Reach (participation rate):** Proportion of the intended priority audience that participates in the intervention; often measured by attendance; includes documentation of barriers to participation				**n = 21**
5.1	**Community activity attendance:** Number or proportion of participants who attended community engagement activities (e.g., forums, group talks, roadshows, health clubs)	Household surveys; structured observations during activities; programmatic data (activity logs, summary reports, attendance records)	[32, 33, 40, 41, 45, 49, 51, 52]	9
5.2	**Participant demographics:** Demographics of participants reached by the intervention (e.g., sex, age, gender)	Household surveys; questionnaire with participants; observations during community meetings; programmatic data (activity logs)	[32, 33, 40, 41, 53]	5
5.3	**Training attendance of community representatives:** Number or proportion of community representatives who attended training (e.g., compound members, community coordinators, and extension workers)	Programmatic data (summary reports); questionnaire with community representatives; structured observations during trainings	[41, 51, 53]	3
5.4	**School activity attendance:** Number or proportion of parents and students who attended school-based events (e.g., hygiene training sessions, drama skits on menstrual hygiene)	Programmatic data (attendance records, observation logs)	[39]	1
5.5	**Student consumable receipt:** Number or proportion of students that received and/or redeemed intervention consumables (e.g., menstrual hygiene kits and painkillers)	Programmatic data (voucher records); questionnaire with recipients	[39]	1
5.6	**Community activity engagement**: Number or proportion of participants engaged in commitment-based activities (e.g., handwashing pledges)	Structured observations during activities	[32]	1
5.7	**Implementers’ presence:** Number or proportion of intervention areas with trained personnel, volunteers, or community representatives in attendance or leading activities (e.g., trained community health workers facilitating group sessions, local volunteers leading hygiene demonstrations, extension agents conducting school-based activities)	Structured observations during activities	[51]	1
**6. Recruitment:** Procedures used to approach and attract participants at individual or organizational levels; includes maintenance of participant involvement in intervention and measurement components of study				**n = 1**
6.1	**Beneficiary registration:** Age of eligible beneficiaries at the start of a program activity (e.g., number of eligible participants, children, registered for nutrition supplements disaggregated by age group)	Programmatic data (data from tracking and registration system)	[38]	1

**Table 4:** Qualitative process indicators used in process evaluations of WASH interventions

#	Qualitative process evaluation questions	Means of verification	Study references	Number of references
**1. Fidelity (quality):** Extent to which intervention was implemented as planned				**n = 9**
1.1	**Activity adherence to design:** Were activities implemented as planned, and how did participants and implementers perceive their content, quality, successes, and challenges?	Interviews with implementers (e.g., staff or volunteers); interviews with intervention recipients (e.g., household members); programmatic documentation (training reports, quality improvement verification checklists); unstructured observations during school and community visits	[32, 35, 41, 43]	4
1.2	**Activity deviation and adaptation:** Were there any deviations from planned activities, and what changes were made to adapt the intervention during implementation?	Interviews with implementers (e.g., staff or volunteers); unstructured observations during community activities; programmatic documentation (activity reports, photographs, field notes)	[40, 41, 46]	3
1.3	**Implementation timeline assessment:** What was the actual timeline of activities, and in what ways did timing affect intervention delivery?	Interviews with implementers (e.g., staff or volunteers); unstructured observations of implementation activities	[43]	1
1.4	**Staff expectations of delivery:** What were the expectations of implementing staff regarding feasibility, usefulness, and likely outcomes of the program?	Interviews with implementers (e.g., staff or volunteers)	[46]	1
**2. Dose delivered (completeness):** Amount or number of intended units of each intervention or component delivered or provided by interventionists				**n = 2**
2.1	**Intervention activity delivery:** How did implementers experience the delivery of key intervention activities (e.g., training sessions, household visits), including factors that influenced how many were conducted, the timeframe of delivery, and which components were most difficult or easiest to deliver?	Interviews with implementers (e.g., staff or volunteers); semi-structured observations of training meetings with implementers	[35, 43]	2
**3. Dose received (exposure):** Extents to which participants actively engage with, interact with, are receptive to, and/or use materials or recommended resources; can include “initial use” and “continued use.”				**n = 4**
3.1	**Interaction with and use of intervention components:** How did participants interact with and use intervention components in their daily lives, and what factors influenced their uptake and continued use)?	Programmatic documentation (implementer debriefs); Interviews with intervention recipients (e.g., household members)	[35, 47, 48]	3
3.2	**Message recall and understanding:** To what extent did participants recall and understand key intervention messages (e.g., at household, community, or school levels), and how did they describe the relevance or usefulness of these messages?	Interviews with implementers (e.g., staff or volunteers); semi-structured observations during community activities and household visits	[43]	1
**4. Dose received (satisfaction):** Participant (primary and secondary audiences) satisfaction with program, interactions with staff and/or investigators				**n = 11**
4.1	**Perceived acceptability and emotional response:** How acceptable were the intervention messages, delivery modes, and activities, and what emotional responses did these evoke (e.g., what was liked or disliked, what made participants feel proud, embarrassed)?	Interviews with implementers (e.g., staff or volunteers); interviews with recipients (e.g., household member, schoolchildren, caregivers); focus group discussions with implementers and recipients; programmatic documentation (e.g., implementer debriefs)	[32, 33, 35, 39–41, 46]	7
4.2	**Preferences and suggested improvements:** What were participants’ preferences regarding intervention delivery (e.g., hygiene program through community events), and what changes would they recommend improving the activities?	Interviews with intervention recipients (e.g., household members); interviews with implementers (e.g., staff or volunteers); programmatic documentation (implementer debriefs)	[33, 35, 46]	3
4.3	**Participant perceptions:** How did participants perceive the intervention (e.g., level of influence in program-related decision-making), and how engaged did they feel (e.g., during participatory design)?	Interviews with recipients (e.g., informal settlement residents); focus group discussions with recipients	[45]	1
**5. Reach (participation rate):** Proportion of the intended priority audience that participates in the intervention; often measured by attendance; includes documentation of barriers to participation				**n = 3**
5.1	**Staff participation across stages:** How did staff participation at each stage of intervention delivery influence how the target population was reached?	Programmatic documentation (session notes, photography, training materials, program reports); semi or unstructured observations of implementers during community activities	[46]	1
5.2	**Target population participation:** In what ways did the target population participate in intervention activities or components?	Programmatic documentation (e.g., session notes and photography); semi or unstructured observations of implementers during community activities	[46]	1
5.3	**Household visit reach:** Which household members were present through household visits?	Programmatic documentation (e.g., debrief sessions with implementers)	[48]	1
**6. Recruitment:** Procedures used to approach and attract participants at individual or organizational levels; includes maintenance of participant involvement in intervention and measurement components of study				**n = 8**
6.1	**Recruitment methods:** What strategies were used to recruit participants into the program, and which approaches worked well or were particularly successful?	Interviews with implementers (e.g., staff or volunteers); programmatic documentation (e.g., reports and debriefing notes); focus group discussions with implementers	[38, 40, 41, 48]	4
6.2	**Recruitment barriers:** What challenges or barriers were encountered during participant recruitment?	Interviews with implementers (e.g., staff or volunteers)	[33, 40, 41]	3
6.3	**Recruitment facilitators**: What successes or facilitators were reported during participant recruitment?	Interviews with implementers (e.g., staff or volunteers); focus group discussions with implementers	[33]	1

Quantitative indicators were most used to assess dose received (exposure), fidelity, dose delivered and reach. These indicators were commonly derived from programmatic data, household surveys, and structured observations. Fidelity indicators assessed whether key intervention components—such as hardware installation, behavior change activities, and materials—were delivered as planned. Dose delivered captured the completeness of implementation, including the number and frequency of training sessions, material distributions, and household visits. Dose received (exposure) reflected participant interaction with the intervention, including message recall and the presence or use of materials such as handwashing stations. Reach was typically assessed through attendance records and demographic data to evaluate inclusivity and coverage. Less frequently reported were quantitative indicators related to dose received (satisfaction) and recruitment, each represented by a single measure across studies.

Qualitative indicators also spanned all domains but were most often used to assess dose received (satisfaction), recruitment, and fidelity. These indicators were typically assessed through interviews, focus group discussions, and reviews of programmatic documentation, enabling deeper exploration of participant and implementer experiences, delivery quality, and factors affecting uptake. These methods surfaced implementation challenges, adaptations, and perceptions of intervention feasibility and acceptability. Dose delivered, dose received (exposure), and reach were less frequently explored through qualitative methods.

## Discussion

This scoping review synthesizes methodologies from 24 process evaluations of WASH interventions in LMICs and identifies several overarching insights that inform how implementation processes have been assessed in the sector. First, many studies drew on conceptual or theoretical frameworks, often adapting them, reflecting the growing use of implementation science approaches within the WASH sector while also highlighting opportunities to apply these frameworks more intentionally to strengthen the methodology of future evaluations. Second, evaluations commonly measured core domains—such as fidelity, dose delivered, dose received (exposure), and reach—using both quantitative and qualitative approaches, while highlighting opportunity for improved measurement of under-assessed domains such as dose received (satisfaction) and recruitment. Third, several studies demonstrated the feasibility of embedding process evaluation activities within routine program structures by leveraging existing monitoring systems, supervision visits, and programmatic data and documentation. Together, these takeaways point to clear opportunities for advancing implementation-focused research and strengthening how process evaluations are designed, executed, and reported in the WASH sector.

### Advancing framework use and improving implementation reporting

The majority of included studies reported using a conceptual or theoretical framework to guide their process evaluation activities [1, 3–5, 7, 26–30]. This reflects the growing integration of implementation science principles in the WASH sector and demonstrates the value practitioners and researchers place on structured approaches for describing, guiding, and evaluating implementation [15]. However, the application of frameworks varied considerably—reflecting broader trends across sectors in which frameworks are often cited but not consistently embedded into study design, data collection, or interpretation [55–57]. For example, while some studies used frameworks conceptually, not all linked them explicitly to indicator development, data types, or data collection timelines, making it difficult to assess alignment between the framework, evaluation design, and implementation process. Reporting of indicators was also inconsistent, with process measures variably presented the methods versus results sections, and limited detail often provided on the means of verification used to assess key domains. These patterns highlight the need for more deliberate and integrated use of frameworks to fully leverage their utility in advancing implementation science in WASH. Key recommendations for applying implementation science—such as selecting a suitable framework, defining research questions, selecting research and evaluation methods, specifying and evaluating implementation outcomes, and reporting findings [58]—are highly relevant for designing and executing robust process evaluations. Adherence to these steps can improve the quality, transparency, and utility of implementation-focused research across WASH programs.

Although implementation reporting in WASH remains variable [13], our findings suggest that many implementation-focused evaluations in the WASH sector have already emphasize documenting what was implemented, how it was delivered, and the extent of participant engagement. The most frequently prioritized domains were fidelity, dose received (exposure), reach, and dose delivered—all of which closely align with key items from the TIDieR-WASH checklist [14]. Although TIDieR-WASH is not a process evaluation framework, its emphasis on documenting core implementation elements corresponds with the types of indicators synthesized in this review. Strengthening routine use of TIDieR-WASH can therefore improve the completeness of implementation reporting, complementing process evaluation efforts. This overlap points to a strong foundation on which to build and highlights an opportunity to further strengthen evaluations of WASH interventions by more consistently measuring and reporting on these key implementation items, thereby enhancing evidence synthesis, adaptation, and policy guidance in the sector.

While our review did not aim to determine which process evaluation approaches lead to specific implementation or health outcomes, the broader implementation science literature in WASH emphasizes that reporting implementation outcomes is a critical foundation for understanding how interventions function in practice [15]. Clear and systematic documentation of implementation can enable future work to examine how different process evaluation approaches relate to implementation performance or effectiveness and to inform decisions about which evaluation tools or frameworks may be most useful across diverse programs.

Similarly, although this review did not assess context because of the variation in how it was defined and reported across studies—a pattern consistent with the broader WASH and global health literature [13, 59]—the frameworks used in the included studies do position context as a core domain of process evaluation. The challenge, however, lies in how these frameworks conceptualize context, as definitions are often very broad and provide limited guidance on what should be assessed. For example, the MRC guidance defines context as “anything external to the intervention that may act as a barrier or facilitator to its implementation, or its effects.” [3, 5] While intentionally flexible, this expansive definition offers little structure for determining which contextual features matter most or for ensuring consistent assessment across studies.

In contrast, an expanding implementation science literature provides more structured tools for conceptualizing and assessing context [60]. The Consolidated Framework for Implementation Research (CFIR), for example, is a determinant framework that identifies barriers and facilitators that may influence implementation, organizing these determinants across domains related to the intervention, the settings in which it is delivered, the individuals involved, and the implementation processes [61]. The Context and Implementation of Complex Interventions (CICI) framework complements CFIR by describing broader external contextual conditions including geographical, epidemiological, sociocultural, socioeconomic, ethical, legal, and political and promotes a multilayered approach to contextual analysis that goes beyond lists of factors [62]. Using an established framework to guide contextual assessment could help future WASH process evaluations identify relevant contextual conditions more consistently, describe how they influence implementation, and strengthen the clarity and usefulness of findings across diverse settings.

### Strengthening measurement across process evaluation domains

Across the included studies, clear patterns emerged in how process evaluation domains were assessed. Evaluations frequently measured fidelity, dose delivered, dose received (exposure), and reach, demonstrating a shared recognition of the importance of documenting what was implemented and how participants engaged with key intervention components. These domains were typically assessed using a mix of quantitative and qualitative approaches. Domains such as dose received (satisfaction) and recruitment were examined less consistently, often relying primarily on qualitative methods. This variation highlights an opportunity to expand and strengthen measurement in under-assessed areas that are central to understanding participant experience, program acceptability, and equitable reach. More balanced assessment across all process evaluation domains would enable a fuller picture of implementation processes and contribute to clearer interpretation of how delivery conditions shape program outcomes.

These findings highlight the complementary strengths of quantitative and qualitative approaches in process evaluations of WASH interventions. Quantitative methods are well suited for assessing implementation scope, consistency, and reach at scale, while qualitative methods offer critical insight into how and why interventions succeed—or fall short—within specific contexts. Aligning data collection methods with evaluation objectives and priority domains can help maximize the utility of available resources and ensure that findings are both meaningful and actionable. The synthesized indicators and examples presented here offer a practical resource to support WASH practitioners and researchers in designing more robust and actionable implementation-focused evaluations.

### Embedding process evaluations within routine program cycles

Several studies demonstrated the feasibility of integrating process evaluation activities directly into routine program structures, highlighting practical opportunities for strengthening implementation learning without substantial additional resources. For example, programmatic data and documentation were commonly used to assess fidelity and dose delivered against established delivery protocols. Household surveys conducted for midline, endline, or annual monitoring typically collect household- and individual-level data related to the intervention’s outcomes [6] and provided efficient platforms for integrating targeted questions on participant exposure, adoption, and perceptions. Field visits, frequently conducted as part of supervisory or participatory monitoring activities in WASH interventions [63–65], provided opportunities for structured observations of intervention delivery. Feedback mechanisms, such as pause-and-reflect sessions, also enabled implementers and recipients to share insights on implementation fidelity, delivery challenges, and contextual factors—providing real-time qualitative process data to inform adaptive management and strengthen program responsiveness [66–68].

These examples demonstrate the potential for process evaluation to be embedded within existing programmatic and evaluation workflows, thereby supporting continuous learning and adaptive management. Integrating process evaluation activities from the outset of program planning can promote timely insights, enhance responsiveness to local conditions, and strengthen implementation quality [16, 17]. Together, these findings point to promising pathways for making implementation-focused evaluation more feasible, sustainable, and actionable within routine WASH programming.

### Strengths and limitations

This scoping review provides a comprehensive overview of how process evaluations have been applied in WASH interventions in LMICs, synthesizing frameworks, methods, data types, and indicators across diverse contexts. One of the primary strengths of this review is its systematic approach to identifying and categorizing quantitative and qualitative indicators across standard process evaluation domains. By highlighting methodological patterns and illustrative examples, this review offers a practical resource for practitioners and researchers seeking to strengthen evaluation design and implementation learning. However, several limitations should be noted. First, studies were included only if they explicitly used the terms ‘process evaluation’ or ‘fidelity’ in their title or abstract. As a result, we may have missed relevant studies that reported implementation-related outcomes without labeling them as process evaluations. This selection criterion may limit the generalizability of findings and underrepresents the full breadth of implementation assessment conducted within WASH programs. Second, practitioner-led process evaluations, particularly those conducted internally within non-governmental organizations, government agencies, or implementing partners, are likely underrepresented, as they may not be published or publicly accessible. Third, the process of extracting and synthesizing process indicator into standardized domains required subjective judgment, particularly when original studies did not explicitly define them. Although we used standard domains to support consistency, the variability in how process evaluation methods were described across studies presented challenges for data extraction and synthesis. Lastly, while this review aimed to catalog a wide range of methodological approaches, it does not assess the quality or completeness of reporting in each study, consistent with the aims of a scoping review. Despite these limitations, our findings underscore the value of applying structured frameworks and quantitative and qualitative approaches in process evaluations and highlight opportunities for applying best practices to assess and report on implementation across WASH programs.

## Conclusion

This scoping review highlights key opportunities to strengthen the design, execution, and reporting of process evaluations in WASH programs. More intentional use of conceptual and theoretical frameworks, coupled with structured reporting tools such as TIDieR-WASH, can enhance the clarity, transparency, and comparability of implementation assessments. This also includes drawing on implementation science frameworks that provide structured approaches for conceptualizing and assessing context. Expanding measurements in under-assessed domains, particularly dose received (satisfaction) and recruitment, will help provide a more complete understanding of how participants experience and engage with interventions. The synthesized indicators presented here offer a practical resource to guide more consistent and actionable measurement across process evaluation domains. In addition, integrating process evaluation activities into routine program cycles by leveraging existing monitoring systems and programmatic tools represents a feasible approach for strengthening real-time learning and adaptive management. Taken together, these strategies can advance implementation-focused research and improve the effectiveness, scalability, and sustainability of WASH interventions across diverse settings.

## Supplementary Information

Additional file 1: Search Strategy.

Additional file 2: Preferred Reporting Items for Systematic reviews and Meta-Analyses extension for Scoping Reviews (PRISMA-ScR) Checklist.

## Data Availability

All data generated or analyzed during this study are included in this published article and its supplementary information files.
