# Supplementary material for: Conducting process evaluations of WASH interventions: a scoping review of design approaches and indicators in low- and middle-income countries
Source: Implement Sci Commun. 2026 Feb 17;7:51. doi: 10.1186/s43058-026-00872-8 (PMC13020263; doi:10.1186/s43058-026-00872-8)
Supplement: Supplementary file 1 — Additional file 1: Search Strategy. [file 43058_2026_872_MOESM1_ESM.docx]

**Search Strategy**

**Searches executed:**

5/15/2024

**Databases:**

1. Ovid Medline - 550
2. Embase - 785
3. Scopus - 1188
4. Web of Science - 1721
5. Global Health - 129

**Ovid Medline (550)**

| **#** | **Search Statement** | **Results** |
| --- | --- | --- |
| 1 | exp Implementation Science/ or exp Health Plan Implementation/ or exp "Process Assessment, Health Care"/ | 12954 |
| 2 | (((process or processes or program*) adj2 (evaluat* or assess* or quality or fidelity)) or ((implement* or interven*) adj2 (evaluat* or assess* or quality or fidelity or research* or science))).ti. | 22098 |
| 3 | 1 or 2 | 33531 |
| 4 | exp Water Quality/ or exp Drinking Water/ or exp Water Purification/ or exp Hygiene/ or exp Water Supply/ or exp Detergents/ or exp Sanitation/ or exp Bathroom Equipment/ or exp Wastewater/ or exp urination/ | 288249 |
| 5 | ("water sanitation and hygiene" or WASH or bore well* or borewell* or open well* or (rainwater adj2 harvest*) or (water adj3 (drinking or unsafe or untreated or access or contaminat* or collect* or chlorin* or clean* or decontaminat* or disinfect* or fetch* or filter* or filtrat* or fluoride or insecurity or potable or purifi* or quality or recontaminat* or safety or source* or storage or supply or treatment)) or detergent* or (hand adj2 (disinfect* or hygien* or sanitiz* or sanitis* or wash*)) or handwash* or soap* or (menstrua* adj2 (hygiene or practice*)) or bathroom or commode or diarrh?ea* or excreta or f?eces or f?ecal or latrine* or lavator* or open defecation or restroom or septic system* or septic tank* or sewage or toilet* or urinat* or urinal or (waste adj2 (dispos* or manage*)) or wastewater).ti,ab,kw,kf. | 624921 |
| 6 | 4 or 5 | 771264 |
| 7 | exp humans/ or exp animals/ | 27185322 |
| 8 | exp humans/ | 21964129 |
| 9 | 7 not 8 | 5221193 |
| 10 | 3 and 6 | 562 |
| 11 | 10 not 9 | 541 |

**Embase (785)**

('implementation science'/exp OR (((process OR processes OR program*) NEAR/2 (evaluat* OR assess* OR quality OR fidelity)):ti) OR (((implement* OR interven*) NEAR/2 (evaluat* OR assess* OR quality OR fidelity OR research* OR science)):ti)) AND ('water quality'/exp OR 'drinking water'/exp OR 'water management'/exp OR 'hygiene'/exp OR 'water supply'/exp OR 'detergent'/exp OR 'sanitation'/exp OR 'bathroom equipment'/exp OR 'wastewater'/exp OR 'micturition'/exp OR (((('water sanitation and hygiene' OR wash OR 'bore well*' OR borewell* OR 'open well*' OR (rainwater NEAR/2 harvest*) OR (water NEAR/3 (drinking OR unsafe OR untreated OR access OR contaminat* OR collect* OR chlorin* OR clean* OR decontaminat* OR disinfect* OR fetch* OR filter* OR filtrat* OR fluoride OR insecurity OR potable OR purifi* OR quality OR recontaminat* OR safety OR source* OR storage OR supply OR treatment)) OR detergent* OR (hand NEAR/2 (disinfect* OR hygien* OR sanitiz* OR sanitis* OR wash*)) OR handwash* OR soap* OR (menstrua* NEAR/2 (hygiene OR practice*)) OR bathroom OR commode OR diarrh?ea* OR excreta OR f?eces OR f?ecal OR latrine* OR lavator* OR open) AND defecation OR restroom OR septic) AND system* OR septic) AND tank*) OR sewage OR toilet* OR urinat* OR urinal OR (waste NEAR/2 (dispos* OR manage*)) OR wastewater) NOT (('human'/exp OR 'animal'/exp) NOT 'human'/exp)

**Scopus (1188)**

( ( INDEXTERMS ( "Implementation Science" OR "Health Plan Implementation" OR "Process Assessment, Health Care" ) OR TITLE ( ( process OR processes OR program* ) W/2 ( evaluat* OR assess* OR quality OR fidelity ) OR ( ( implement* OR interven* ) W/2 ( evaluat* OR assess* OR quality OR fidelity OR research* OR science ) ) ) ) AND ( INDEXTERMS ( "Water Quality" OR "Drinking Water" OR "Water Purification" OR "Hygiene" OR "Water Supply" OR "Detergents" OR "Sanitation" OR "Bathroom Equipment" OR "Wastewater" OR "urination" OR "water management" OR "detergent" OR "micturition" ) OR TITLE-ABS-KEY ( ( "water sanitation and hygiene" ) OR wash OR "bore well*" OR borewell* OR "open well*" OR ( rainwater W/2 harvest* ) OR ( water W/3 ( drinking OR unsafe OR untreated OR access OR contaminat* OR collect* OR chlorin* OR clean* OR decontaminat* OR disinfect* OR fetch* OR filter* OR filtrat* OR fluoride OR insecurity OR potable OR purifi* OR quality OR recontaminat* OR safety OR source* OR storage OR supply OR treatment ) ) OR detergent* OR ( hand W/2 ( disinfect* OR hygien* OR sanitiz* OR sanitis* OR wash* ) ) OR handwash* OR soap* OR ( menstrua* W/2 ( hygiene OR practice* ) ) OR bathroom OR commode OR diarrh?ea* OR excreta OR f?eces OR f?ecal OR latrine* OR lavator* OR open AND defecation OR restroom OR septic AND system* OR septic AND tank* OR sewage OR toilet* OR urinat* OR urinal OR ( waste W/2 ( dispos* OR manage* ) ) OR wastewater ) ) ) AND NOT INDEXTERMS ( ( human OR humans OR animal OR animals ) AND NOT ( human OR humans ) )

**Web of Science (1721)**

Indexes=SCI-EXPANDED, SSCI, A&HCI, CPCI-S, CPCI-SSH, BKCI-S, BKCI-SSH, ESCI, CCR-EXPANDED, IC Timespan=All years

((TI=((process OR processes OR program*) NEAR/2 (evaluat* OR assess* OR quality OR fidelity) OR ((implement* OR interven*) NEAR/2 (evaluat* OR assess* OR quality OR fidelity OR research* OR science)))) AND TS=(("water sanitation and hygiene") OR WASH OR "bore well*" OR borewell* OR "open well*" OR (rainwater NEAR/2 harvest*) or (water NEAR/3 (drinking or unsafe or untreated or access or contaminat* or collect* or chlorin* or clean* or decontaminat* or disinfect* or fetch* or filter* or filtrat* or fluoride or insecurity or potable or purifi* or quality or recontaminat* or safety or source* or storage or supply or treatment)) OR detergent* OR (hand NEAR/2 (disinfect* or hygien* or sanitiz* or sanitis* or wash*)) or handwash* OR soap* OR (menstrua* NEAR/2 (hygiene or practice*)) OR bathroom OR commode OR diarrh?ea* OR excreta OR f?eces or f?ecal OR latrine* or lavator* OR open defecation OR restroom or septic system* OR septic tank* OR sewage OR toilet* OR urinat* OR urinal OR (waste NEAR/2 (dispos* or manage*)) OR wastewater))

**Global Health (129)**

[[et: "process evaluation"] OR [et: "process assessment"] OR [et: "process quality"] OR [et: "process fidelity"] OR [et: "processes evaluation"] OR [et: "processes assessment"] OR [et: "processes quality"] OR [et: "processes fidelity"] OR [et: "program evaluation"] OR [et: "program assessment"] OR [et: "program quality"] OR [et: "program fidelity"] OR [et: "implementation evaluation"] OR [et: "implementation assessment"] OR [et: "implementation quality"] OR [et: "implementation fidelity"] OR [et: "implementation research"] OR [et: "implementation science"] OR [et: "intervention evaluation"] OR [et: "intervention assessment"] OR [et: "intervention quality"] OR [et: "intervention fidelity"] OR [et: "intervention research"] OR [et: "intervention science"]] AND [[All: "water sanitation and hygiene"] OR [All: wash] OR [All: "bore well"] OR [All: "bore wells"] OR [All: borewell*] OR [All: "open well"] OR [All: "open wells"] OR [All: "rainwater harvesting"] OR [All: "harvested rainwater"] OR [All: "harvesting rainwater"] OR [All: "drinking water"] OR [All: "unsafe water"] OR [All: "untreated water"] OR [All: "water access"] OR [All: "water contamination"] OR [All: "water contaminant"] OR [All: "contaminated water"] OR [All: "water collection"] OR [All: "water collecting"] OR [All: "collecting water"] OR [All: "chlorinated water"] OR [[All: water] AND [All: chlorine]] OR [All: "water cleaning"] OR [All: "water decontamination"] OR [All: "water disinfection"] OR [All: "water fetching"] OR [All: "fetching water"] OR [All: "water filter"] OR [All: "filtered water"] OR [All: "water filters"] OR [All: "water filtration"] OR [[All: water] AND [All: fluoride]] OR [All: "water insecurity"] OR [All: "potable water"] OR [All: "water purification"] OR [All: "water quality"] OR [All: "water recontamination"] OR [All: "water re-contamination"] OR [All: "water safety"] OR [All: "water source"] OR [All: "water storage"] OR [All: "water supply"] OR [All: "water treatment"] OR [All: detergent*] OR [All: "hand disinfection"] OR [All: "hand sanitization"] OR [All: "hand sanitisation"] OR [All: "hand wash"] OR [All: "hand washing"] OR [All: handwash*] OR [All: soap*] OR [All: "menstrual hygiene"] OR [All: "menustrual practices"] OR [All: bathroom] OR [All: commode] OR [All: diarrhea*] OR [All: diarrhoea*] OR [All: excreta] OR [All: feces] OR [All: faeces] OR [All: fecal] OR [All: faecal] OR [All: latrine*] OR [All: lavator*] OR [All: "open defecation"] OR [All: restroom] OR [All: "septic system"] OR [All: "septic systems"] OR [All: "septic tank"] OR [All: "septic tanks"] OR [All: sewage] OR [All: toilet*] OR [All: urinat*] OR [All: urinal] OR [All: "waste disposal"] OR [All: "waste management"] OR [All: wastewater]]

**Supplemental Table 1.** Summary of process evaluation studies included in the scoping review.

| **Study** | **Country** | **Intervention summary and approach** | **Demand-side** | **Supply-side** | **Process evaluation aim and methods** |  | **Quantitative methods** | Household survey | Structured observation | Questionnaire | Programmatic data | Non-programmatic data | **Qualitative methods** | Interviews | Focus groups | Semi /unstructured observation | Programmatic documentation |
| --- | --- | --- | --- | --- | --- | --- | --- | --- | --- | --- | --- | --- | --- | --- | --- | --- | --- |
| **WASH category** – **Water: Interventions aimed at improving access to or microbiological quality of drinking water** | | | | | | | | | | | | | | | | | |
| Barstow, 2016 [1] | Rwanda | ***Tubeho Neza “Live Well” Program*** - Provided water filters, cookstoves, and education and behavior change messaging to promote adoption and use of water filters and cookstoves in rural communities/households | **✓** | **✓** | Assess adoption and sustained use of filters and cookstoves, monitor maintenance and repairs, and gather program data |  | **✓** | + |  |  | + |  |  |  |  |  |  |
| Bradshaw 2021 [2] | Rwanda | ***Gikuriro “Well- Growing Child” Program* -** Integrated water filter distribution and training; supported health club facilitators to educate and assist households in filter use | **✓** | **✓** | Describe integration of best practices for filter distribution and promotion with a health messaging program, measured by coverage and use |  | **✓** | + |  |  | + |  |  |  |  |  |  |
| Gallandat, 2024 [3] | Democratic Republic of the Congo | ***Urban Piped Water Supply Infrastructure Improvement Program*** - Upgraded treatment plant and pumping station, added storage tank, and installed or rehabilitated pipes and taps to improve water supply and quality for urban households |  | **✓** | Assess validity of the program’s theory of change across context, implementation, and population response domains |  | **✓** | + |  |  |  | + | **✓** |  |  |  | + |
| **WASH category – Sanitation: Interventions aimed at improving access to or use of safe sanitation and feces management** | | | | | | | | | | | | | | | | | |
| Boisson, 2014 [4] | India | ***Total Sanitation Campaign (TSC)*** - Provided latrine subsidies to low-income rural households and used community mobilization and information, education and communication activities to encourage latrine adoption and use | **✓** | **✓** | Describe the intervention context, document delivery, assess exposure among the target population, and explore the link between community mobilization and latrine construction within a cluster-randomized trial |  | **✓** | + | + | + | + |  | **✓** | + |  |  | + |
| Irani, 2021 [5] | India | ***JEEViKA Technical Support Program*** - Trained community mobilizers in health, nutrition, and sanitation topics to share knowledge through self-help group meetings and home visits targeted for mothers of young children in rural areas | **✓** |  | Capture strategic, structural, and system-level changes reported by mobilizers after training |  | **✓** |  |  | + |  |  |  |  |  |  |  |
| **WASH category – Hygiene: Interventions aimed at promoting hygiene behaviors (e.g., handwashing at key moments, menstrual and food hygiene practices)** | | | | | | | | | | | | | | | | | |
| Divya Rajaraman, 2014 [6] | India | ***SuperAmma*** - Promoted handwashing using non-health messages through a communication campaign in rural communities, households, and schools, primarily targeting mothers and children | **✓** |  | Assess implementation and influence on social norms and perceived benefits |  | **✓** | + | + |  |  |  | **✓** | + |  | + |  |
| Greenland, 2017 [7] | Zambia | ***Komboni Housewives Intervention*** - Tested a strategy using affiliation and disgust to motivate caregivers to adopt four diarrhea control practices, including handwashing in rural and peri-urban areas | **✓** |  | Investigate how delivery and receipt factors influenced caregiver uptake of target behaviors |  | **✓** | + | + |  | + |  | **✓** | + | + |  |  |
| Linabarger, 2018 [8] | Kenya | ***Care Group Model Adaptation in Western Kenya*** - Trained local volunteers to promote behavior change, extending reach beyond CHWs, and building capacity of pregnant women, mothers of children <2, and health centers in rural areas | **✓** |  | Assess fidelity of Care Group model delivery and identify factors influencing implementation to improve behavior change messaging |  |  |  |  |  |  |  | **✓** | + |  | + | + |
| Ruel-Bergeron, 2019 [9] | Malawi | ***Large-Scale Community-Based Nutrition Program*** - Provided nutrient supplements and behavior change communication to improve infant and young child feeding and WASH practices in rural households with children 6–23 months | **✓** |  | Measure recruitment, reach, and fidelity based on the program’s implementation theory |  | **✓** |  | + | + | + |  | **✓** |  |  |  | + |
| Waterkeyn, 2020 [10] | Rwanda | ***Community Health Club Application in Rwanda*** - Trained rural communities via Health Clubs to promote hygiene behavior change; used certificates and membership cards to encourage participation and track attendance | **✓** |  | Assess training effectiveness and influence of contextual factors on intermediate outcomes in the cluster-randomized controlled trial |  | **✓** | + |  |  | + |  | **✓** |  | + |  |  |
| Simiyu, 2023 [11] | Kenya | ***‘Safe Start’ Trial*** - Delivered food hygiene behavior change intervention via household visits by community health volunteers, targeting key caregiver practices of children under five in a peri-urban settlement. | **✓** |  | Document intervention delivery, understand delivery context, and explore links between exposure and adoption |  | **✓** | + |  |  | + |  | **✓** | + |  |  | + |
| Thorseth, 2023 [12] | Zimbabwe | ***Wash’Em Process Application in Zimbabwe*** - Applied Wash’Em process to design evidence-based handwashing programs for drought-affected populations | **✓** |  | Document implementation of each Wash’Em phase in a crisis setting, guided by a theory of change to inform program design |  |  |  |  |  |  |  | **✓** | + | + | + | + |
| **WASH category – Combined: Interventions aimed at integrating two or more WASH components** | | | | | | | | | | | | | | | | | |
| Chard & Freeman, 2018 [13] | Lao People’s Democratic Republic | ***UNICEF’s Laos WinS Project*** - Delivered WASH facilities and promoted health and hygiene behaviors in primary schools | **✓** | **✓** | Assess intervention fidelity, including delivery per objectives and adherence by teachers and students at school level |  | **✓** |  | + | + |  |  |  |  |  |  |  |
| Antwi-Agyei, 2017 [14] | Tanzania | ***Tanzania National Sanitation Campaign (NSC)*** - Provided water sources, improved latrines, established school health clubs, developed hygiene materials, trained officials, and led monitoring and advocacy efforts in rural areas | **✓** | **✓** | Assess whether NSC was implemented as intended per theory of change, and if inputs led to expected behavior and output changes |  | **✓** |  | + |  |  |  | **✓** | + |  |  | + |
| Gelli, 2019 [15] | Burkina Faso | ***SELEVER+WASH Study*** - Integrated a poultry-livestock perspective into the Community-Led Total Sanitation (CLTS) behavior change approach which included creation of village hygiene committees in rural areas | **✓** |  | Assess fidelity of implementation, characterize activities and service quality, and identify implementation challenges and opportunities |  | **✓** | + |  |  |  |  | **✓** | + | + |  | + |
| Bhuyian, 2020 [16] | Bangladesh | ***Cholera-Hospital-Based Intervention for 7-days (CHoBI7)*** ***Mobile Health (mHealth) Program*** - Delivered mobile messages to households of diarrhea patients promoting water treatment and handwashing | **✓** |  | To assess the fidelity, dose, and reach of the CHoBI7 mHealth program in delivering mHealth messages during a randomized controlled trial. |  | **✓** |  |  | + | + |  |  |  |  |  |  |
| D'Mello-Guyett, 2020 [17] | Democratic Republic of Congo | ***Médecins Sans Frontières 2018 Cholera Outbreak Response***- Distributed hygiene kits that included water treatment products and a handwashing device, and demonstrated their use to patients and their accompanying household members upon admission to health facilities in rural areas | **✓** | **✓** | Assess implementation, participant response, and context to identify successes, challenges, and recommendations for hygiene kit distribution in cholera control |  | **✓** |  | + |  | + | + | **✓** | + |  |  | + |
| Nalugya, 2020 [18] | Uganda | ***Menstrual Health Interventions and School Attendance in Uganda (MENISCUS) Intervention*** - Delivered teacher training, drama skits, menstrual kit use training, pain relief guidance, and improved school WASH facilities | **✓** | **✓** | Describe pilot implementation quality, participation, and acceptability; assess context and mechanisms; and discuss implications for scale-up |  | **✓** |  | + | + | + |  | **✓** | + | + |  |  |
| Bick, 2021 [19] | Mozambique | ***MapSan Trial*** - Provided improved pour-flush toilets with shared septic tank, handwashing sink, and hygiene promotion by community organizations to compounds in urban areas | **✓** | **✓** | Assess fidelity (dose received, reach), participant response, and context 24–42 months post-intervention |  | **✓** |  | + | + |  |  |  |  |  |  |  |
| Ramanadhan, 2022 [20] | India | ***Tuver Health & Wellness Centre Project*** - Delivered a multi-component intervention to support cross-sector change and improve health, including running water and community toilets in rural areas |  | **✓** | Examine context and partnership activities during early intervention assessment |  | **✓** |  |  |  | + |  | **✓** |  |  |  | + |
| Sclar, 2022 [21] | India | ***“Sundara Grama” Intervention*** - Delivered multi-level communication at community, group, and household levels, promoting the motto 'My Clean, Healthy, Beautiful Village’ targeting caregivers of children under five, community members and leaders in rural areas | **✓** | **✓** | Quantitatively assess fidelity, dose delivered, and reach; qualitatively examine recruitment, context, and satisfaction |  | **✓** | + | + |  | + |  | **✓** | + |  | **+** | + |
| Freeman, 2022 [22] | Ethiopia | ***“Andilaye” Intervention*** - Delivered multi-level communication at district, community, group, and household levels, promoting the motto 'Together we can be a strong, caring, healthy community’ in rural and peri-urban areas | **✓** |  | Describe and analyze dose delivery, participation, and dose received to inform program impacts |  | **✓** | + | + | + | + |  |  |  |  |  |  |
| Panulo, 2022 [23] | Malawi | ***“Hygienic Family” Intervention*** - Delivered WASH and food hygiene intervention through household visits and caregiver groups using peer education and behavior change techniques to reduce diarrheal disease among children under five in rural communities | **✓** |  | Assess fidelity, dose, reach, acceptability, and impact to inform considerations for intervention scale-up |  | **✓** | + | + |  | + |  | **✓** | + | + |  |  |
| Mink, 2024 [24] | Indonesia and Fiji | ***Revitalising Informal Settlements and their Environments (RISE) Trial*** *-* Implemented water-sensitive infrastructure, including pressure tanks, constructed wetlands, and rainwater tanks to reduce fecal exposure and improve ecological health in urban communities/households |  | **✓** | Evaluate participation, barriers to participation, and participant satisfaction in RISE activities |  | **✓** | + |  |  |  |  | **✓** | + | + |  |  |

**Supplemental Table 2.** Conceptual and theoretical frameworks used in process evaluation of WASH interventions.

| **Framework** | **n (%)** | **Studies** | **Summary of conceptual or theoretical application** | **Explicit application for indicator development and results organization** |
| --- | --- | --- | --- | --- |
| Medical Research Council (MRC) Guidance^25,26^ | 8 (33%) | [3] [7] [11] [12] [16] [18] [19] [23] | The framework was used to report process evaluation outcomes such as fidelity, dose delivered, dose received, reach, acceptability, and contextual factors influencing implementation. | Several studies organized indicators and results using MRC domains (n=7), and some combined MRC with other frameworks (n=5). One study referenced the MRC framework conceptually without applying it to indicator development or structuring results (n=1). |
| Process Evaluation for Public Health Interventions and Research^27^ | 7 (29%) | [4] [7] [9] [11][12] [19] [23] | This framework was used to report domains such as fidelity, dose delivered, dose received, reach, recruitment, and context. | Multiple studies used the framework to guide the organization of indicators and reporting by domains (n=6), while one study applied it primarily as a conceptual guide without explicitly shaping indicator development or results organization (n=1). |
| A Conceptual Framework for Implementation Fidelity^28^ | 3 (13%) | [13] [19] [22] | The framework was applied to assess fidelity of implementation, including adherence, moderating factors, and influence on outcomes. | Where used, this framework guided the development and presentation of fidelity indicators (n=2), although one study referenced the framework without clearly applying it to indicator development or structuring results (n=1). |
| Developing a Process-Evaluation Plan for Assessing Health Promotion Program Implementation: A How-To Guide^29^ | 3 (13%) | [8] [21] [24] | The framework was used to examine fidelity, reach, dose delivered, dose received, and context in implementation assessments. | All studies guided by this framework (n=3) applied it to develop indicators and organize the results section. |
| Other Frameworks:  Exploration, Preparation, Implementation, Sustainment (EPIS) Framework;^30^ WHO’s Process Evaluation Workbook;^31^ Conceptual models for implementation outcomes and generalizability^32–34^ | 4 (17%) | [7] [11] [13] [20] | Additional frameworks were used to explore adoption, sustainability, and implementation challenges, including system-level and cross-sector considerations. | Some studies used these frameworks to organize findings or integrate domains with other frameworks (n=3). However, in these studies, the explicit influence on indicator development or results organization was variable and generally limited. |

**References**

1. Barstow, C., Nagel, C., Clasen, T. & Thomas, E. Process evaluation and assessment of use of a large scale water filter and cookstove program in Rwanda. *BMC public health* **16**, (16 July 2016).

2. Bradshaw, A., Mugabo, L., Gebremariam, A., Thomas, E. & Macdonald, L. Integration of household water filters with community-based sanitation and hygiene promotion—a process evaluation and assessment of use among households in rwanda. *Sustainability (Switzerland)* **13**, 1–22 (2021).

3. Gallandat, K. *et al.* Process evaluation of an urban piped water supply infrastructure improvement programme in Uvira, Democratic Republic of the Congo. *PLOS Water* **3**, e0000185 (2024).

4. Boisson, S. *et al.* Promoting latrine construction and use in rural villages practicing open defecation: process evaluation in connection with a randomised controlled trial in Orissa, India. *BMC research notes* **7**, 486 (2014).

5. Irani, L., Schooley, J. & Chaudhuri, I. Layering of a health, nutrition and sanitation programme onto microfinance-oriented self-help groups in rural India: results from a process evaluation. *BMC public health* **21**, (2021).

6. Divya Rajaraman, D. R. *et al.* Implementing effective hygiene promotion: lessons from the process evaluation of an intervention to promote handwashing with soap in rural India. *BMC public health* **14**, (19 November 2014) (2014).

7. Greenland, K., Chipungu, J., Chilekwa, J., Chilengi, R. & Curtis, V. Disentangling the effects of a multiple behaviour change intervention for diarrhoea control in Zambia: a theory-based process evaluation. *Global Health* **13**, 78 (2017).

8. Linabarger, M. A qualitative process evaluation of maternal and baby WASH and nutrition project using a Care Group approach in western Kenya. (Emory University, Rollins School of Public Health, 2018).

9. Ruel-Bergeron, J. C. *et al.* Process Evaluation of a Large-Scale Community-Based Nutrition Program in Malawi. *Current developments in nutrition* **4**, nzz131 (2020).

10. Waterkeyn, J. *et al.* The value of monitoring data in a process evaluation of hygiene behaviour change in Community Health Clubs to explain findings from a cluster-randomised controlled trial in Rwanda. *BMC public health* **20**, 98 (2020).

11. Simiyu, S. *et al.* A Mixed Methods Process Evaluation of a Food Hygiene Intervention in Low-Income Informal Neighbourhoods of Kisumu, Kenya. *Maternal and child health journal* **27**, 824–836 (2023).

12. Thorseth, A. H. *et al.* Using Wash’Em to Design Handwashing Programmes for Crisis-Affected Populations in Zimbabwe: A Process Evaluation. *International journal of environmental research and public health* **21**, (2024).

13. Chard, A. & Freeman, M. Design, Intervention Fidelity, and Behavioral Outcomes of a School-Based Water, Sanitation, and Hygiene Cluster-Randomized Trial in Laos. *International journal of environmental research and public health* **15**, 570 (2018).

14. Antwi-Agyei, P. *et al.* Water, sanitation and hygiene (WASH) in schools: Results from a process evaluation of the national sanitation campaign in Tanzania. *Journal of Water Sanitation and Hygiene for Development* **7**, 140–150 (2017).

15. Gelli, A. *et al.* Examining the implementation of multisectoral programs: the SELEVER process evaluation. *IFPRI - Discussion Papers* vi + 47 pp.

16. Bhuyian, M. S. I. *et al.* Process evaluation for the delivery of a water, sanitation and hygiene mobile health program: findings from the randomised controlled trial of the CHoBI7 mobile health program. *Tropical Medicine and International Health* **25**, 985–995 (2020).

17. D’Mello-Guyett, L. *et al.* Distribution of hygiene kits during a cholera outbreak in Kasai-Oriental, Democratic Republic of Congo: a process evaluation. *Conflict and health* **14**, 51 (2020).

18. Nalugya, R. *et al.* Assessing the effectiveness of a comprehensive menstrual health intervention program in Ugandan schools (MENISCUS): process evaluation of a pilot intervention study. *Pilot and feasibility studies* **6**, (2020).

19. Bick, S. *et al.* Using path analysis to test theory of change: a quantitative process evaluation of the MapSan trial. *BMC public health* **21**, 1411 (2021).

20. Ramanadhan, S., Ganapathy, K., Nukala, L., Rajagopalan, S. & Camillus, J. C. A model for sustainable, partnership-based telehealth services in rural India: An early process evaluation from Tuver village, Gujarat. *PloS one* **17**, (2022).

21. Sclar, G. D. *et al.* Mixed Methods Process Evaluation of a Sanitation Behavior Change Intervention in Rural Odisha, India. *Glob Implement Res Appl* **2**, 67–84 (2022).

22. Freeman, M. C. *et al.* The impact of a demand-side sanitation and hygiene promotion intervention on sustained behavior change and health in Amhara, Ethiopia: A cluster-randomized trial. *PLOS Global Public Health* **2**, e0000056 (2022).

23. Panulo, M. *et al.* Process Evaluation of ‘The Hygienic Family’ Intervention: A Community-Based Water, Sanitation, and Hygiene Project in Rural Malawi. *International journal of environmental research and public health* **19**, (2022).

24. Mink, T. L. *et al.* Who participates in ‘participatory design’ of WASH infrastructure: a mixed-methods process evaluation. 2024.06.12.24308866 Preprint at https://doi.org/10.1101/2024.06.12.24308866 (2024).

25. Craig, P. *et al.* Developing and evaluating complex interventions: the new Medical Research Council guidance. *BMJ* **337**, a1655 (2008).

26. Skivington, K. *et al.* A new framework for developing and evaluating complex interventions: update of Medical Research Council guidance. *bmj* **374**, (2021).

27. Steckler, A. E. & Linnan, L. E. *Process Evaluation for Public Health Interventions and Research.* (Jossey-Bass/Wiley, 2002).

28. Carroll, C. *et al.* A conceptual framework for implementation fidelity. *Implementation Science* **2**, 40 (2007).

29. Saunders, R. P., Evans, M. H. & Joshi, P. Developing a process-evaluation plan for assessing health promotion program implementation: a how-to guide. *Health Promot Pract* **6**, 134–147 (2005).

30. EPIS Framework. *EPIS Framework* https://episframework.com.

31. WHO. *Process Evaluations Workbook*. (2000).

32. Grant, A., Treweek, S., Dreischulte, T., Foy, R. & Guthrie, B. Process evaluations for cluster-randomised trials of complex interventions: a proposed framework for design and reporting. *Trials* **14**, 15 (2013).

33. Bonell, C., Oakley, A., Hargreaves, J., Strange, V. & Rees, R. Assessment of generalisability in trials of health interventions: suggested framework and systematic review. *Bmj* **333**, 346–349 (2006).

34. Proctor, E. *et al.* Outcomes for implementation research: conceptual distinctions, measurement challenges, and research agenda. *Adm Policy Ment Health* **38**, 65–76 (2011).
